# Effect of a Profound Feedstock Change on the Structure and Performance of Biogas Microbiomes

**DOI:** 10.3390/microorganisms8020169

**Published:** 2020-01-25

**Authors:** Johanna Klang, Ulrich Szewzyk, Daniel Bock, Susanne Theuerl

**Affiliations:** 1 Leibniz Institute for Agricultural Engineering and Bioeconomy, Max-Eyth-Allee 100, 14469 Potsdam, Germany; dbock@atb-potsdam.de (D.B.); susanne.theuerl@googlemail.com (S.T.); 2 Department of Environmental Microbiology, Technische Universität Berlin, Ernst-Reuter-Platz 1, 10587 Berlin, Germany; ulrich.szewzyk@tu-berlin.de

**Keywords:** anaerobic digestion, energy crops, manure, ammonium, terminal restriction fragment length polymorphism (TRFLP), indicator taxa

## Abstract

In this study the response of biogas-producing microbiomes to a profound feedstock change was investigated. The microbiomes were adapted to the digestion of either 100% sugar beet, maize silage, or of the silages with elevated amounts of total ammonium nitrogen (TAN) by adding ammonium carbonate or animal manure. The feedstock exchange resulted in a short-range decrease or increase in the biogas yields according to the level of chemical feedstock complexity. Fifteen taxa were found in all reactors and can be considered as generalists. Thirteen taxa were detected in the reactors operated with low TAN and six in the reactors with high TAN concentration. Taxa assigned to the phylum *Bacteroidetes* and to the order *Spirochaetales* increased with the exchange to sugar beet silage, indicating an affinity to easily degradable compounds. The recorded TAN-sensitive taxa (phylum *Cloacimonetes*) showed no specific affinity to maize or sugar beet silage. The archaeal community remained unchanged. The reported findings showed a smooth adaptation of the microbial communities, without a profound negative impact on the overall biogas production indicating that the two feedstocks, sugar beet and maize silage, potentially do not contain chemical compounds that are difficult to handle during anaerobic digestion.

## 1. Introduction

The demand and use of renewable energy such as biogas have risen during the last decades because anaerobic digestion is attributed with several advantages such as a sustainable waste management or the integration into multi-faceted production systems for food, feed, bioenergy, and biomaterials [1] and because of the negative climate impact of fossil fuels. 

Biogas is the end-product of the anaerobic digestion of organic matter. It consists mostly of methane (CH_4_) and carbon dioxide (CO_2_), whereas the energy rich CH_4_ is the desired compound. For the production of biogas, different kinds of organic matter can be used, such as wastewater sludge, municipal solid waste, residues from livestock husbandry, and energy crops. According to the Renewable Energy Act in 2000, especially with the amendments in 2004 and 2009, most agricultural biogas plants in Germany are operated with energy crops and livestock residues [1,2]. The most used energy crop is maize, as it is easy to cultivate and has high biomass and biogas yields. Consequently, the cultivation of maize was expanded [3,4]. Although it is not fully quantified, negative effects of the Renewable Energies Act became obvious and led to, for example, the food versus fuel debate e.g., [5]. Moreover, the expanded cultivation of energy crops such as maize has several negative impacts, such as the loss of biodiversity and reduced soil fertility as well as soil degradation. In the last couple of years, several amendments of the Renewable Energy Source Act have been made. In 2012, an upper maize limit of 60% was introduced which was further reduced to 50% in 2017 [5,6]. However, the transition to a circular bioeconomy is required, in which anaerobic digestion (biogas production) is a keystone for providing base load power which can be supplied on demand and serves as a sustainable residue management strategy [1]. Manifold residues from agricultural crop production, livestock husbandry, and landscape management, as well as from organic wastes from food processing, food consumption, and from the organic fraction of municipal solid waste might be appropriate feedstocks for anaerobic digestion [1]. Consequently, plant operators will be forced to use a broad range of different feedstocks which are chemically very heterogeneous, variable over time, and often available only in small quantities. This will lead to a more inhomogeneous feedstock supply and sometimes to rapid feedstock changes, which might cause problems for the microbial community in the biogas reactors as the available nutrients and the bioaccesibility may differ [1,7]. 

The production of biogas is a complex process where several different microorganisms using different metabolic pathways work together to degrade organic matter into biogas. The process can be divided into four main steps: the hydrolysis, the acidogenesis, the acetogenesis, and the methanogenesis. The first three steps are conducted by anaerobic bacteria which gradually digest organic matter into mainly acetic acid, CO_2_, and hydrogen (H_2_). The methanogenesis is performed by methanogenic archaea, which convert the end products of the first three steps into biogas. Because of the low energy amount gained under anaerobic conditions, one organism cannot perform a complete digestion; therefore, several bacteria work together to digest the feedstock into the substrate used by the methanogens [8,9,10]. This leads to a high diversity on the bacterial level, with up to several thousand different operational taxonomic units (OTUs) in one biogas plant [11,12]. The development of the microbial community depends on the available substrates. Different feedstocks with different chemical compositions and bioavailability lead to different microbial communities [11,13,14,15,16,17,18,19]. Therefore, knowledge of the microbial reaction to different feedstock compositions is highly important in order to avoid process disturbances, and changes in the feedstock composition have to be performed with caution. One well known factor leading to process disturbances are elevated concentrations of ammonium in equilibrium with ammonia [7,20]. Ammonium/ammonia is produced during degradation of nitrogenous compounds, cannot be further degraded under anaerobic conditions, and accumulates in the reactors. The amount of ammonia depends on the pH value and temperature; the higher these values, the more ammonia occurs in the system. It has been shown that ammonia is the cause of process inhibition [7,20,21] as it can diffuse through the cell wall causing proton imbalance or potassium deficiency [21]. Furthermore, it has been shown that the acetoclastic archaeal genus *Methanothrix* is the most sensitive one against elevated ammonia concentrations, and that, for example, *Methanoculleus* or *Methanosarcina* tolerate higher concentrations [22]. 

The objective of this study was to analyze the reaction of biogas microbiomes to a profound feedstock change from a chemically more complex to an easily degradable feedstock and vice versa. Prior to this study, the microbiomes were adapted to the digestion of either 100% sugar beet or maize silage, of 100% sugar beet or maize silage with increasing amounts of ammonium carbonate, or to the co-digestion of 25% silage and 75% animal manure. Based on these preconditions, we suspected (i) that the exchange of chemically different feedstocks will cause a significant change (increase or decrease) in the biogas yields, (ii) that the microbial communities will change in their taxonomic composition, whereby it is of interest how long they will need to adapt to the new conditions, (iii) that the effects will be lowest for the reactor system where only 25% of the feedstock mixture was exchanged, and (iv) that microorganisms or groups of microorganisms can be identified which have an affinity to either sugar beet or maize silage, or which do tolerate elevated TAN concentrations. In order to elucidate these hypotheses, the fingerprint method terminal restriction fragment length polymorphism (TRFLP) in combination with a cloning/sequencing approach was used to monitor the dynamics of the bacterial and archaeal communities, which was further related to the occurring/changing environmental conditions using multivariate statistical analyses.

## 2. Materials and Methods 

### 2.1. Reactor Operation

For the eight-week study, six continuously stirred tank reactors (CSTRs) with a working volume of three liters were used. Prior to this study, the reactors had been operated under different conditions for approximately three years. The reactors were adapted to the digestion of either sugar beet (SB) or maize (M) silage as a single feedstock, to the digestion of sugar beet or maize silage with increasing amounts of ammonium carbonate, or to co-digestion of either sugar beet or maize silage and animal manure [14]. In this study, the feedstock supply was changed, meaning that the reactors operated with sugar beet silage were given maize silage and vice versa. This means:

- the reactor formerly fed with sugar beet silage was now given maize silage (SB-M1),
- the reactor formerly fed with maize silage was now given sugar beet silage (M-SB1),
- the reactor formerly fed with sugar beet silage and ammonium carbonate was now given maize silage and ammonium carbonate (SB-M2),
- the reactor formerly fed with maize silage with ammonium carbonate was now given sugar beet silage and ammonium carbonate (M-SB2),
- the reactor formerly fed with 25% sugar beet silage and 75% animal manure was now given 25% maize silage with 75% animal manure (SB-M3), and
- the reactor formerly with 25% maize silage and 75% animal manure was now given 25% sugar beet silage with 75% animal manure (M-SB3).

The total ammonium nitrogen (TAN) concentration in the reactors SB-M1 and M-SB1 were kept at 0.3 g·L^−1^ through the addition of ammonium carbonate, whereas the TAN concentration in the reactors SB-M2 and M-SB2 were adapted to the concentrations in the reactors given manure (SB-M3 and M-SB3) twice a week. The trace element solution DMSZ 144 (Leibniz Institute DSMZ-German Collection of Microorganisms and Cell Cultures GmbH, Braunschweig, Germany) was added to every feeding in order to avoid process inhibition through lack of micronutrients according to Elhussein and Weiland [23]. The reactors were operated under mesophilic conditions (40 °C) and fed once a day. The organic loading rate (OLR) was 2.0 g_·_L^−1^·d^−1^ with a hydraulic retention time (HRT) of 43 days. The biogas was collected in gasbags (Tesseraux Spezialverpackungen GmbH, Bürstadt, Germany). The produced biogas amount was measured at least twice a week using a drum-type gas meter (Dr.-Ing. Ritter Apparatebau GmbH & Co. KG, Bochum, Germany) while the gas composition was analyzed using the portable analyzer “BiogasCheck” (Geotechnical Instruments Ltd. UK, Coventry, United Kingdom). Samples for chemical and microbiological analyses were taken at least once a week. 

### 2.2. Chemical Analyses

The pH value was measured prior to feeding. The contents of total solids (TS), volatile solids (VS), volatile fatty acids (VFA, acetic, propionic, (i)-butyric, (i)-valeric, and caproic acids), total Kjeldahl nitrogen (TKN), and total ammonium nitrogen (TAN) were analyzed according to Schönberg and Linke [24].

### 2.3. Molecular Biological Analyses

The DNA was extracted as triplicate using the DNeasy PowerSoil Kit (Qiagen, Hilden, Germany). The DNA was amplified with PCR according to Klang et al. [13] using the primer pair 27f/926Mr for the bacterial community and 109f/912r for the archaeal community. As the PCR product should be analyzed using TRFLP, the forward primer of each assay was labeled with Cy5. The PCR products were purified using the Nucleospin Gel and PCR Clean-up (Macherey und Nagel, Düren, Germany). A restriction digestion was performed with the endonucleases MspI and Hin6I for the bacterial community and AluI for the archaeal community. The samples were analyzed using the capillary sequencer GenomeLab™ GeXP Genetic Analysis System (AB SCIEX Germany GmbH, Darmstadt, Germany) according to Rademacher et al. [25]. The phylogenetic assignment of the recorded terminal restriction fragments (TRFs) was conducted with the sequence library produced in Klang et al. [13,14]. Additionally, we used an in-house database, currently containing about 3000 16S rRNA gene sequences from various projects carried out at the Leibniz Institute for Agricultural Engineering and Bioeconomy (ATB) during the last ten years [19]. Within these projects, the microbial community structure in laboratory- and full-scale biogas reactors was investigated. All sequences within this database were obtained by the same protocols (e.g., nucleic acid extraction, analyzed variable 16S rRNA gene region, and especially the bioinformatic data evaluation) which increases/ensures the correct assignment of the recorded TRFs. Briefly, after DNA extraction, PCR amplification of the 16S rRNA gene was carried out using the same primer pairs (this time unlabeled) and PCR conditions as mentioned for the TRFLP analysis. Cloning of purified 16S rRNA gene amplicons was performed according to Rademacher et al. [25], while DNA sequencing was conducted by Eurofins Genomics (Eurofins Genomics GmbH, Ebersberg, Germany). After a quality check of the obtained sequences, a multiple alignment was performed using the Needleman-Wunsch algorithm with a CLUSTW similarity calculation in combination with an UPGMA clustering using the Kimura-2-parameter correction (BioNumerics 7.6, Applied Maths, Kortrijk, Belgium). Alignment sequences were grouped into operational taxonomic units (OTUs) at 94.5% and 98.65% sequence similarity for the genus and species level, respectively [26,27]. The OTUs were phylogenetically identified using the RDP Naïve Bayesian rRNA Classifier Version 2.11 (Michigan State University, East Lansing, MI, USA) [28] and virtually digested as previously described by Klang et al. [13].

### 2.4. Bioinformatics and Statistical Analysis

The obtained electropherograms were analyzed using the software package BioNumerics (Applied Maths, Kortrijk, Belgium) according to Klang et al. [13].

Only TRFs with a relative abundance over 1% were used for the statistical analysis. The calculation of the Richness as well as the Shannon diversity indices was performed with the R Project for Statistical Computing [29] using the packages “vegan” [30]. In order to assess the community evenness, the Gini index based on the calculation of the normalized area between the a Lorenz curve compared to a perfect evenness line [31,32] was calculated using the R package “ineq” [33]. Non-metric multidimensional scaling (NMDS) [34] was also performed with the R Project for Statistical Computing [29] using the packages “vegan” [30]. The distance matrix was calculated using the Bray-Curtis algorithm [35]. Environmental vectors were calculated using “envfit”, while the results were sorted according to the highest R^2^ values as the ten first vectors are shown. To identify TRFs which are characteristic for specific environmental conditions, we calculated an indicator species analysis (ISA) according to Dufréne and Legendre [36] using the “indicspecies” package of R [37].

## 3. Results and Discussion

### 3.1. Chemical and Operational Parameters

In this study, the response of a profound feedstock change on the reactor performance and the microbial community was investigated. Prior to this change, the reactors had been operated for 911 days, first with sugar beet silage or maize silage as a single feedstock for about one year (until day 337) [13], followed by a gradual exchange/addition of either animal manure or ammonium carbonate (until day 911) [14]. 

At the beginning of this study, which was started at day 918 (after sampling), the two reactors, which had only been operated with silages since the experimental start-up, had an ammonium concentration below 0.3 g·L^−1^. As both sugar beet silage and maize silage are rather nitrogen-poor feedstocks, ammonium carbonate had to be added to avoid process disturbances due to a potential nutrient limitation [13]. In the reactors given ammonium carbonate or animal manure, the TAN concentrations were significantly higher with values of around 4 g·L^−1^, whereas ammonia was around 340 mg·L^−1^. This ammonium concentration is considered to be critically high, as values around 3.0 g·L^−1^ often lead to reactor instabilities [20,21]. However, the microbial communities in the investigated reactor systems were adapted to this TAN concentration [14]. The TAN concentrations were kept constant in all reactors throughout the whole experimental phase.

**Figure 1:**
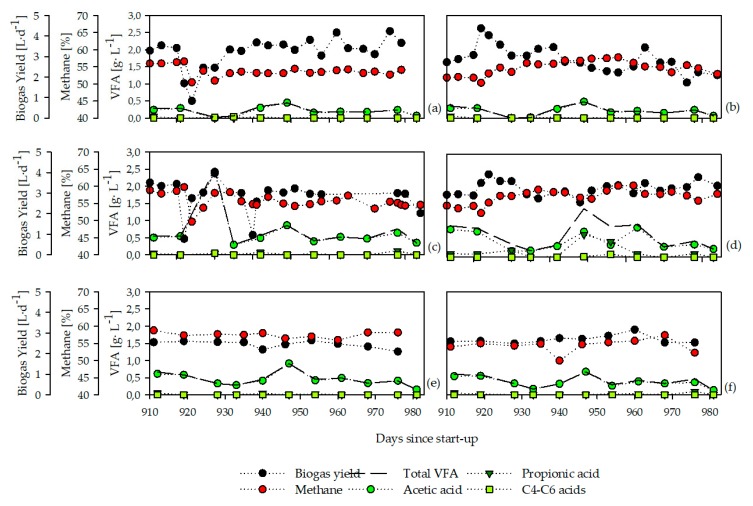
Biogas yields, methane contents, and volatile fatty acids (VFA) of the investigated reactor systems: reactor formerly fed with sugar beet silage now given maize silage (SB-M1) (**a**), reactor formerly fed with maize silage now given sugar beet silage (M-SB1) (**b**), reactor formerly fed with sugar beet silage and ammonium carbonate now given maize silage and ammonium carbonate (SB-M2) (**c**), reactor formerly fed with maize silage with ammonium carbonate now given sugar beet silage and ammonium carbonate (M-SB2) (**d**), reactor formerly fed with 25% sugar beet silage and 75% animal manure now given 25% maize silage with 75% animal manure (SB-M3) (**e**), and reactor formerly with 25% maize silage and 75% animal manure now given 25% sugar beet silage with 75% animal manure (M-SB3) (**f**) during a period of eight weeks.

The biogas yields, methane contents, and volatile fatty acid concentrations over the course of time are shown in Figure 1. Directly after the feedstock change from sugar beet silage to maize silage in the reactors SB-M1 and SB-M2, the biogas yields decreased rapidly but increased again after only some days (Figure 1a,c). In contrast, increasing biogas yields were noted in the reactors M-SB1 and M-SB2 directly after the change from maize silage to sugar beet silage (Figure 1b,d). In the reactors SB-M3 and M-SB3, the reactors fed with 75% chicken manure and only 25% silages, no notable changes in the biogas yields were observed (Figure 1e,f). 

The most probable explanation for the decreasing or increasing biogas yields is the different chemical composition of sugar beet silage and maize silage. The sugar beet silage contains higher amounts of easily degradable compounds, such as sugars, ethanol, and acetic acid while maize silage has larger amounts of more complex compounds, such as cellulose, starch, and hemicellulose, which is related to the kinetics of the biogas production rate in high temporal resolution (hours) for both feedstocks [13]. Hence, the microbial community adapted to the conversion of sugar beet silage needed some time to hydrolyze the more complex compounds in the maize silage into the substrates for the acido-/acetogenesis, and furthermore, the methanogenesis while the community given the easily degradable sugar beet silage instead of the maize silage could convert the given compound more or less directly into biogas shortly after addition. 

Considering the accumulated VFAs, a notable change took place only in the reactors given ammonium carbonate (Figure 1c,d). In the reactor SB-M2, the concentration of acetic acid increased to 2.5 g·L^−1^ some days after the feedstock change, indicating that the bacterial community was able to hydrolyze and ferment the supplied feedstock compounds, but the methanogenic community could not utilize the produced acid at the same speed. However, the concentration decreased within one week, indicating a putative quick adaptation of the archaeal community. Comparatively, in the reactor M-SB2, an accumulation of propionic acid was noted approximately 30 days after the feedstock exchange, whilst the acetic acid concentration decreased. Apparently, compounds in the sugar beet silage were degraded into propionic acid but the community was not capable of further conversion into biogas. This can be explained by the fact that methanogenic archaea only metabolizes acetic acid, H_2_, and one-carbon (C1) compounds; hence, fatty acids such as propionic acid first have to be converted into these utilizable compounds until biogas can be produced in syntrophic relationships [38,39,40]. However, the VFA concentration in the silage reactors (SB-M1, M-SB1) was below 0.5 g·L^−1^ (Figure 1a,b) and below 1.0 g·L^−1^ in the manure reactors (Figure 1e,f) throughout the whole eight weeks.

### 3.2. Bacterial Community - General Overview

The microbial community was investigated using TRFLP in combination with a cloning/sequencing approach. A total number of 150 different bacterial TRFs were found in all investigated reactors. Out of them, 15 TRFs (ca. 10%) were found in all six reactors at least at more than one time point with an overall median abundance of 31% (Figure S1). Among them, TRF 84, 90, 92, 98, and 187 could be assigned to the phylum *Bacteroidetes*, TRF 167 and 218 to the phylum *Firmicutes*, and TRF 163 to the phylum *Thermotogae*; the TRFs 123, 129, 156, 228, 236, 373, and 538 remained unassigned. Calusinska et al. [11] reported that the core microbiome contained several members from the phylum *Bacteroidetes* and *Firmicutes,* which is confirmed by several previous studies reporting high abundances of these two phyla in reactors producing biogas [41,42,43,44,45,46]. In accordance with previous studies, the TRF-related microorganisms seem to be able to exist under various environmental conditions as the six reactor systems differed in their TAN concentrations as well as in the degradability of the supplied feedstocks; hence, they might be putative generalists belonging to the biogas core microbiome [11,19].

**Figure 2:**
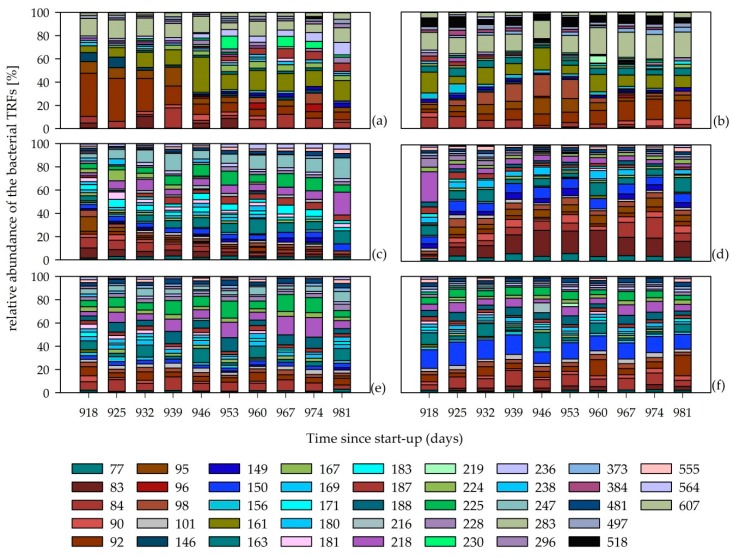
Structure of the bacterial community in reactors SB-M1 (**a**), M-SB1 (**b**), SB-M2 (**c**), M-SB2 (**d**), SB-M3 (**e**), and M-SB3 (**f**). Colored bars symbolize the detected terminal restriction fragments (TRFs) in base pairs (bp) and their relative abundance. For the bacterial community, only TRFs with a relative abundance over 3% in at least one sample are shown. Each sampling point is given as a median value of three technical replicates. Numbers in sampling point descriptors indicate the duration of continuous fermentation in days since start-up.

**Figure 3:**
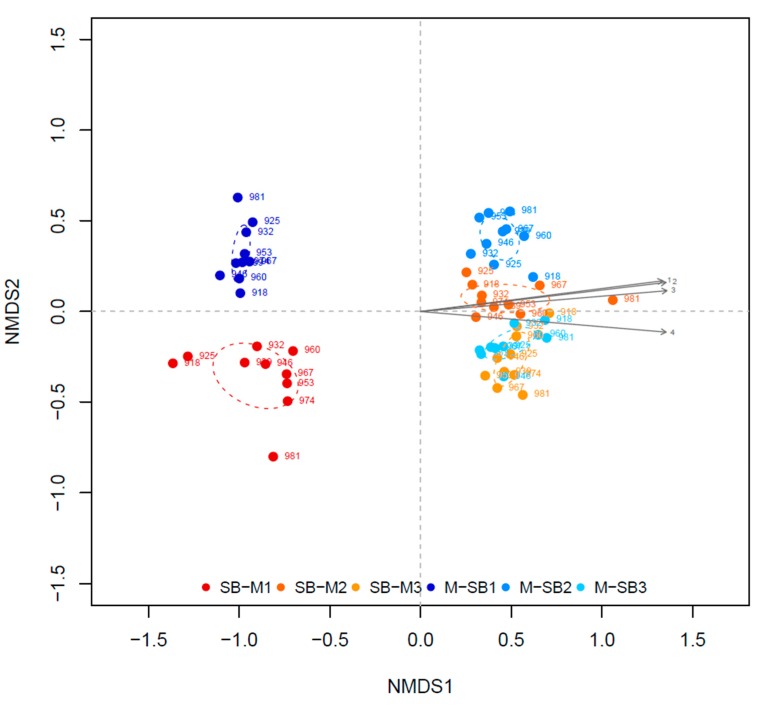
Non-metric dimensional scaling (NMDS) of all six reactors (stress = 0.14). The environmental vectors symbolize 1) total Kjeldahl nitrogen (TKN), 2) total ammonium nitrogen (TAN), 3) ammonia nitrogen (NH_3_), as well as 4) pH value, *p* = 0.001, R^2^ > 0.9.

Opposite to these generalists, several TRFs were found only in certain reactors and seem to be specialists that occupy particular ecological niches or fulfill specific ecosystem functions as they are, for example, important for the degradation of specific substrates [19]. Thirteen TRFs were almost exclusively detected in the reactors operated with low TAN concentrations (Figure S2), while twelve of them were assigned as significant by ISA (Table S1). In comparison, six TRFs mainly occurred in the reactors with high TAN concentrations (Figure S3). Additionally, the ISA analysis revealed that also TRF 150 and TRF 218 are significant for the reactors with high TAN concentration due to their higher relative abundance within these reactors. The TRFLP profiles (Figure 2) as well as the NMDS ordination (Figure 3) clearly distinguish the reactor systems with low (SB-M1 and M-SB1) and high TAN concentration (SB-M2, M-SB2, SB-M3, M-SB3). 

Additionally, the community evenness and richness of the respective communities of the reactors differed: the mean values of the Gini indices were 0.44 ± 0.09 and 0.45 ± 0.08 for SB-M1 and M-SB1, respectively, over the course of time, meaning that the communities in the reactors with low TAN concentrations were more unevenly distributed then the communities in the reactors with high TAN concentrations (SB-M2: 0.37 ± 0.05, SB-M3: 0.33 ± 0.06, M-SB2: 0.41 ± 0.06, M-SB3: 0.38 ± 0.04). Both a high and low Gini index is assumed to be negative considering the ability of the occurring community to be resistant/resilient to sudden stress exposure as both community organizations might need time to adapt to changes; a too unevenly organized community might need time to recover after a change, whereas a too evenly organized community might need time to react to changes caused by a lack of selective pressure; a “good” value is considered to be around 0.45 [31,32,47]. Both reactors given ammonium carbonate (SB-M2, M-SB2) had lower Gini indices compared to the reactors with low TAN concentrations (SB-M1, M-SB1); hence, the recorded VFA accumulation is supposed to be related to unfavorable community organization. The reactors given animal manure showed the lowest values, but no indication for a process instability/disturbance was observed. This might be related to the fact that in both reactors, only 25% of the feedstocks were changed, which most likely was less important for a community adapted to the degradation of 75% animal/chicken manure.

### 3.3. Bacterial Community at Low TAN Concentration

The bacterial community in SB-M1 (Figure 2a) was clearly dominated by TRF 92, assigned to the phylum *Bacteroidetes*, in the beginning of the experimental phase but its relative abundance decreased over the course of time. The reactor M-SB1 (Figure 2b) showed an opposite behavior: the relative abundance of TRF 92 increased over the course of time. Hence, this TRF disappeared as the sugar beet silage was changed into maize silage and increased as sugar beet silage was introduced. These findings further confirm the assumption that certain members of the phylum *Bacteroidetes* have a high affinity to easily degradable compounds (sugars, alcohols, and organic acids) and hence play a crucial role during the acido- and acetogenesis [13,19,48,49,50,51].

Out of the 12 significant TRFs, in the reactors with low TAN concentrations, only two of these TRFs could be phylogenetically assigned: TRF 161 to the not yet cultivatable phylum *Cloacimonetes* and TRF 283 to the phylum *Spirochaetes*. Both TRF 161 and TRF 283 showed high relative abundance with values between 5% and 22% as well as 3% and 18%, respectively, whereas the relative abundances of the other characteristic TRFs were below 5% at all time points. 

Members of the phylum *Cloacimonetes* can be assumed to be sensitive to TAN as it has been reported that their abundance decreases or even disappears at elevated TAN concentrations [14,52,53]. Considering this, the occurrence of members from the phylum *Cloacimonetes* in these reactor systems was expected. Additionally, there was no significant trend towards an increasing or decreasing relative abundance of TRF 161 over the course of time indicating that the TRF-related microorganisms had no specific affinity to maize silage or sugar beet silage.

The nearest neighbor assignable to TRF 283 had a similarity of only 93% to the next known species and can therefore only be assigned to the order *Spirochaetales.* Regarding their functional/ecological role within the biogas microbiome, less information is available. Gupte et al. [54] summarized that members of this order can be anaerobic, facultative anaerobic, or microaerophilic while they perform a chemoorganotrophic metabolism using carbohydrates or amino acids as carbon and energy sources. In the reactors with low TAN concentrations, the relative abundance of TRF 283 showed a tendency to increase in the reactor where the feedstock was changed from maize silage to sugar beet silage, and to decrease when the change was the other way around. Hence, the TRF-related microorganisms seem to have a higher affinity to more easily degradable compounds. However, the relative abundance of TRF 283 in reactor SB-M1 increased again at the last sampling point, so that one possible assumption might be that the newly adapted community was able to degrade the maize silage into compounds suitable for the *Spirochaetales*. 

**Figure 4:**
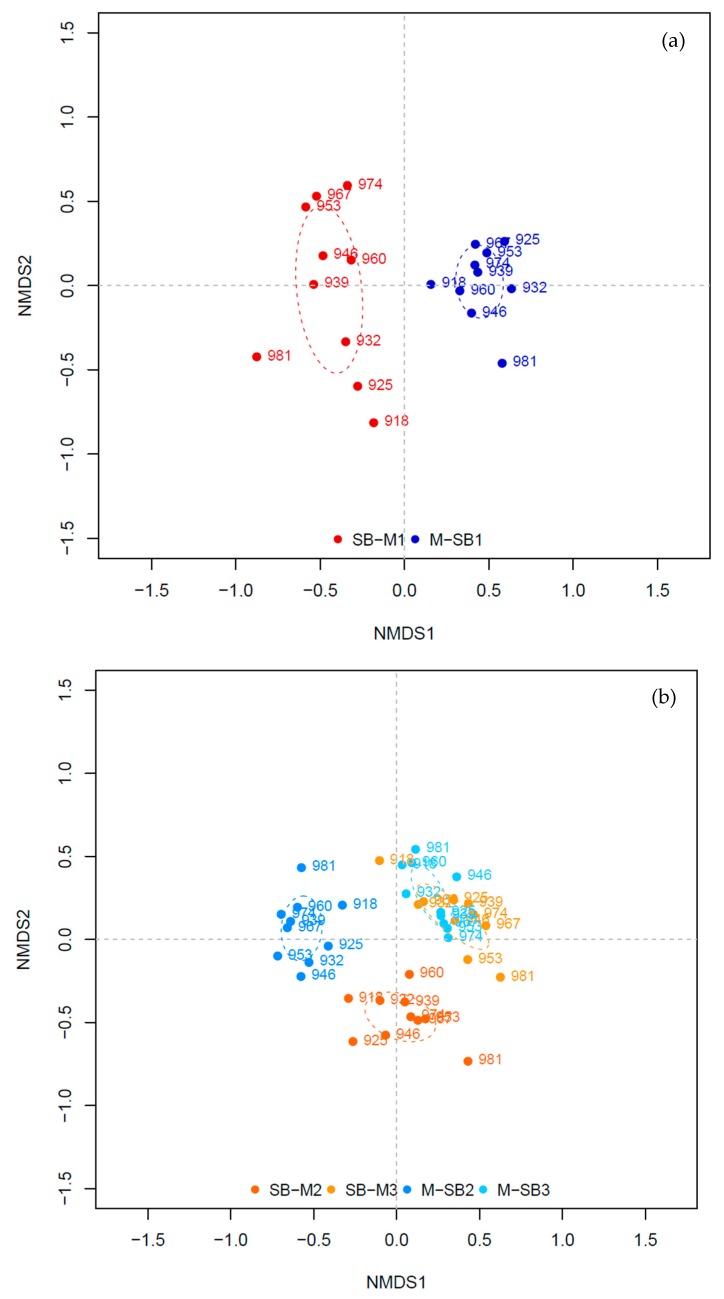
Non-metric dimensional scaling (NMDS) of the reactors with low TAN (**a**), stress = 0.15 and with high TAN (**b**), stress = 0.20.

A comparison of the bacterial communities only in the reactors SB-M1 and M-SB1 revealed that the bacterial community in reactor SB-M1 showed more dynamic variations/changes over time than the bacterial community in reactor M-SB1 (Figure 4a). The similarity between the first and the last sampling days (918 and 981) was 40% in reactors SB-M1 and 60% in M-SB1. The bacterial community in reactor SB-M1 became more diverse over the experimental phase (Figure 2a, Figure S4). This shifted the community composition in the reactor, where sugar beet silage was exchanged with maize silage is most likely caused by the higher amount of chemically more complex compounds within the maize silage, which requires a higher functional and ecological diversity [7,13,19,50,55]. This assumption is further confirmed as three of the SB-M1 characteristic TRFs (Table S2) could be assigned to the phylum *Firmicutes* (TRF 180, 210, and 296), which members are known to be mainly involved during the hydrolysis stage were chemically complex feedstock compounds such as cellulose are primarily degraded [7,9,50,56,57]. Compared to this, among the characteristics TRFs for the M-SB1 community, TRF 163 and 518 (Table S2) could phylogenetically be assigned. TRF 163 could be assigned to the genus *Mesotoga*, the only known mesophilic genus of the phylum *Thermotogea* which is either assumed to be able to degrade different sugars with acetate, carbon dioxide, and sulfide as end-products [58], or which is supposed to be involved in syntrophic acetate degradation [59]. TRF 518 was assigned to the phylum *Chloroflexi* and further to the class *Anaerolineae*. An omic-based genome interpretation showed that members of this class are able to ferment carbohydrates with acetate as an end-product, possibly live in syntrophic relationships, are capable of cell adhesion, and that some members are capable to hydrolyze cellulose [60]. 

Although a profound change in the feedstock regime was performed in this study, which generally should be carried out with caution [7], the reported findings thus far showed a smooth adaptation of the bacterial communities, without a long lasting negative impact on the overall biogas production. This indicates that the two feedstocks, sugar beet and maize silage, potentially do not contain chemical compounds that are difficult to handle during anaerobic digestion [1].

### 3.4. Bacterial Community at High TAN Concentration

Although there was a high dissimilarity between the reactors with low and high TAN concentrations, some similarities were observed considering the bacterial phyla. As already mentioned, the microbial community of reactor SB-M1 was dominated by the TRF 92, assigned to the phylum *Bacteroidetes*, at the beginning of the experimental phase. Comparatively, the reactor SB-M2 showed a high dominance of members from the phylum *Bacteroidetes*, whereby the community was not dominated by one TRF but several different TRFs could be assigned to this phylum (e.g., TRF 84, 92, 95, 96, 98). Similar to reactor SB-M1, the relative abundance of these TRFs decreased over the course of time as the community adapted to the degradation of maize silage (Figure 2a,c). In those reactors where the feedstock was changed from maize to sugar beet silage (M-SB1 and M-SB2), it was the other way around as the relative abundance of TRFs assigned to the phylum *Bacteroidetes* increased over the course of time. The ISA showed that the TRFs 77, 101, 224, 481, 534, and 555 were exclusively found (Figure S3) and most significant (Table S1) for the community with high TAN concentration while none of these TRFs showed a relative abundance higher than 5% (Figure S3). Only TRF 77 could be properly assigned to the order *Syntrophobacterales* (phylum *Proteobacteria)* and showed a sequence similarity of 96% to the species *Syntrophus aciditrophicus.* This species is able to degrade fatty acids and aromatic acids in a syntrophic relationship with hydrogenotrophic methanogens [61]. This finding is in accordance with previous studies which showed a predominance of the hydrogenotrophic pathway of methane formation under elevated TAN concentration e.g., [14,15,38,62,63]. Hydrogenotrophic archaea are also known as hydrogen scavengers, meaning they depend on H_2_ which is produced by their neighboring bacteria (such as members of the genera *Syntophomonas*, *Syntrophobacter*, *Syntrophus*, *Propionibacter*, *Pelotomaculum*, *Smithella,* or *Clostridium* [38,39]) who in turn can only grow if the H_2_ is consumed. Consequently, an inhibition of the function of hydrogenotrophic archaea by NH_4_^+^/NH_3_ can lead to a process disturbance which is reflected not only in reduced methane yields, but also in acid accumulation as it was recorded in reactors SB-M2 and M-SB2.

The NMDS analysis of the four different reactors with high TAN concentrations showed three different clusters: (I) reactor SB-M2, (II) reactor M-SB2, and (III) the two manure reactors (SB-M3 and M-SB3) (Figure 4b). The most significant TRF for cluster I, the SB-M2 community, was TRF 285 that could be assigned to *Aminobacterium colombiense* (phylum *Synergistetes*) with a sequence similarity of 99% [64,65]. Compared to this, the ISA pointed five most significant TRFs for the reactor systems with high TAN concentrations towards cluster II (TRF 79, 104, 143, 554, and 579), reactor M-SB2 (Table S2), while none of them could be phylogenetically assigned to any known species. The two reactors operated with 75% chicken manure (cluster III) showed no significant community change over the course of time indicating that a change of 25% of a “process-uncritical” feedstock did not lead to profound effects on the microbial diversity and hence the overall reactor performance. However, the calculated ISA values for the reactors with high TAN concentrations are generally lower compared to the ISA values for the reactors with low TAN concentrations (Table S2), indicating that the TAN concentration has a higher impact on the community composition (and hence ecosystem function) than the availability of different chemical compounds provided by the supplied feedstocks.

### 3.5. Archaeal Community

**Figure 5:**
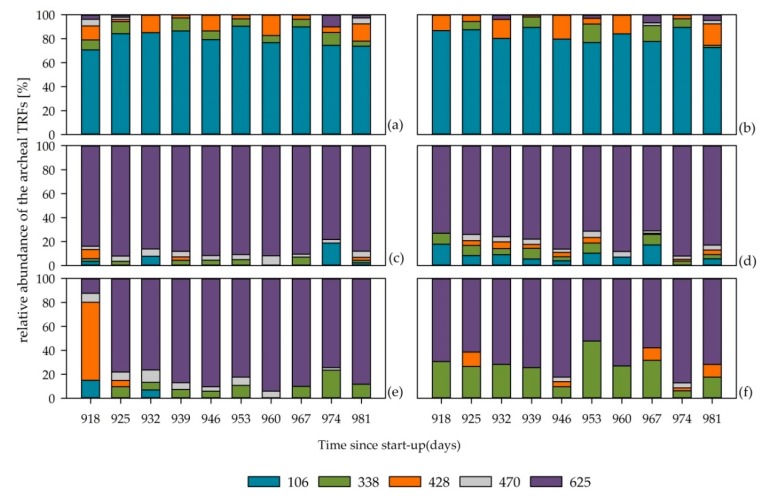
Structure of the archaeal community in reactors SB-M1 (**a**), M-SB1 (**b**), SB-M2 (**c**), M-SB2 (**d**), SB-M3 (**e**), and M-SB3 (**f**). Colored bars symbolize the detected terminal restriction fragments (TRFs) in base pairs (bp) and their relative abundance. Each sampling point is given as a median value of three technical replicates. Numbers in sampling point descriptors indicate the duration of continuous fermentation in days since start-up.

The archaeal communities remained more or less unchanged in the six investigated reactor systems throughout the whole experimental phase (Figure 5).

The archaeal communities in the two reactors with low TAN concentrations were dominated by TRF 106 (Figure 5a,b), which was assigned to the genus *Methanothrix* (order *Methanosarcinales*). This genus is obligate acetoclastic; hence, the members of this genus only produce methane from acetic acid [22,66]. According to the assumption that this genus is abundant in well-performing reactor systems [14,15,67], the VFA concentrations in the reactors SB-M1 and M-SB1 were low while the biogas yields and methane contents were constant, indicating an undisturbed process. Therefore, it can be concluded that the bacterial communities which adapted to the exchanged feedstocks over the course of time were able to degrade the provided chemical compounds into acetic acid, while the archaeal community was not inhibited but could further degrade the intermediates into methane. 

The reactors with high TAN concentrations were dominated by TRF 625 throughout the whole experimental phase (Figure 5c–f). This TRF was assigned to the genus *Methanosarcina,* of which members are able to convert both acetic acid as well as H_2_ and CO_2_ into biogas. Although this genus dominated the communities in reactor SB-M2 and M-SB2, the VFA accumulation (Figure 1c,d) indicated a disturbance in the reactors operated with only silages and ammonium carbonate. One possible explanation for this disturbance could be that changes in the bacterial communities forced the occurring members of the genus *Methanosarcina* to change their pathways of methane formation [22]. As an acetic acid accumulation occurred in reactor SB-M2, it can be assumed that the *Methanosarcina* community changed from the hydrogenotrophic to the acetoclastic pathway. In the reactor M-SB2, an accumulation of propionic acid was observed which might indicate a change from the acetoclastic to the hydrogenotrophic pathway. Hence, it can be assumed that the bacterial community in reactor M-SB2 produced metabolites, in this case propionic acid, which can only be further converted in syntrophic relationships, whereas the bacterial community in reactor SB-M2 degraded the supplied feedstock into high amounts of acetic acid that is further degraded into biogas by acetoclastic methanogens. This assumption was already discussed by Klang et al. [14] and is strengthened by the observations in this study.

Besides the predominance of the genus *Methanosarcina*, in the reactor SB-M3, in which 25% of the feedstock composition was changed from sugar beet silage to maize silage, TRF 428, assigned to *Methanoculleus*, showed a high relative abundance at the first sampling day. Since *Methanoculleus* produces methane via the hydrogenotrophic pathway, it was assumed that also *Methanosarcina* used this pathway, at least in the beginning of the experimental phase. Since no acid accumulation occurred, it is not possible to conclude whether the acetoclastic or the hydrogenotrophic pathway was used throughout the experimental phase. 

## 4. Conclusions

Different microorganisms have different nutrient requirements and hence different degradation capacities to certain feedstock compounds. This study showed that the chemical complexity of the supplied feedstocks (either various compounds or complex molecule structures) directly affects the diversity of the biogas microbiome and hence the process performance. As expected, the exchange of maize and sugar beet silage against each other resulted in a short-term decrease or increase of the biogas yields according to the chemical feedstock complexity, which could be related to a change in the taxonomic composition of the occurring microbial communities. Regarding the hypotheses, this study revealed that certain members of the phylum *Bacteriodetes*, as well as members of the order *Spirochaetales*, have an affinity to the conversion of easily degradable compounds, indicating their involvement in the acido-/acetogenesis. Moreover, this study confirmed previous findings that members of the yet not cultivated phylum *Cloacimonetes* (symbolized by TRF 161) are sensitive to elevated TAN concentration as the respective TRF was only recorded within the reactor systems with low TAN concentrations, while no significant trend towards an increasing or decreasing relative abundance in relation to the feedstock exchange was detected.

Although established methods, such as TRFLP which was used in this study, are still of great value to study the microbial community dynamics over time in relation to the impact of changing environmental conditions, the disadvantage is the putatively low phylogenetic resolution which hamper a distinct identification of specific microbial process indicators. However, the results give further valuable information in order to better understand the biogas microbiome response to changing environmental conditions which are putatively risky for the process performance. With respect to the current methodological development and an urgent need to identify and verify microbial process indicators which are usable for monitoring the dynamic process of biogas production, new sequencing technologies, such as Nanopore sequencing, are expected to offer the possibility to capture the microbial diversity with high phylogenetic resolution down to the species level.

## Supplementary Materials

The following are available online at https://www.mdpi.com/2076-2607/8/2/169/s1, Figure S1: Relative abundance of the twelve TRFs found in all six reactors at least at more than one time point, Figure S2: Relative abundance of the 13 TRFs found mainly in the reactors with low TAN concentration, Figure S3: Relative abundance of the seven TRFs found mainly in the reactors with high TAN concentration. Figure S4: Shannon diversity indices for the microbial communities of all six reactors over the experimental phase. Table S1: Indicator species analysis.
