# Supplementary material for: Effect of a Profound Feedstock Change on the Structure and Performance of Biogas Microbiomes
Source: Microorganisms. 2020 Jan 25;8(2):169. doi: 10.3390/microorganisms8020169 (PMC7074709; doi:10.3390/microorganisms8020169)
Supplement: Supplementary file 1 [file microorganisms-08-00169-s001.pdf]

Supplemental information

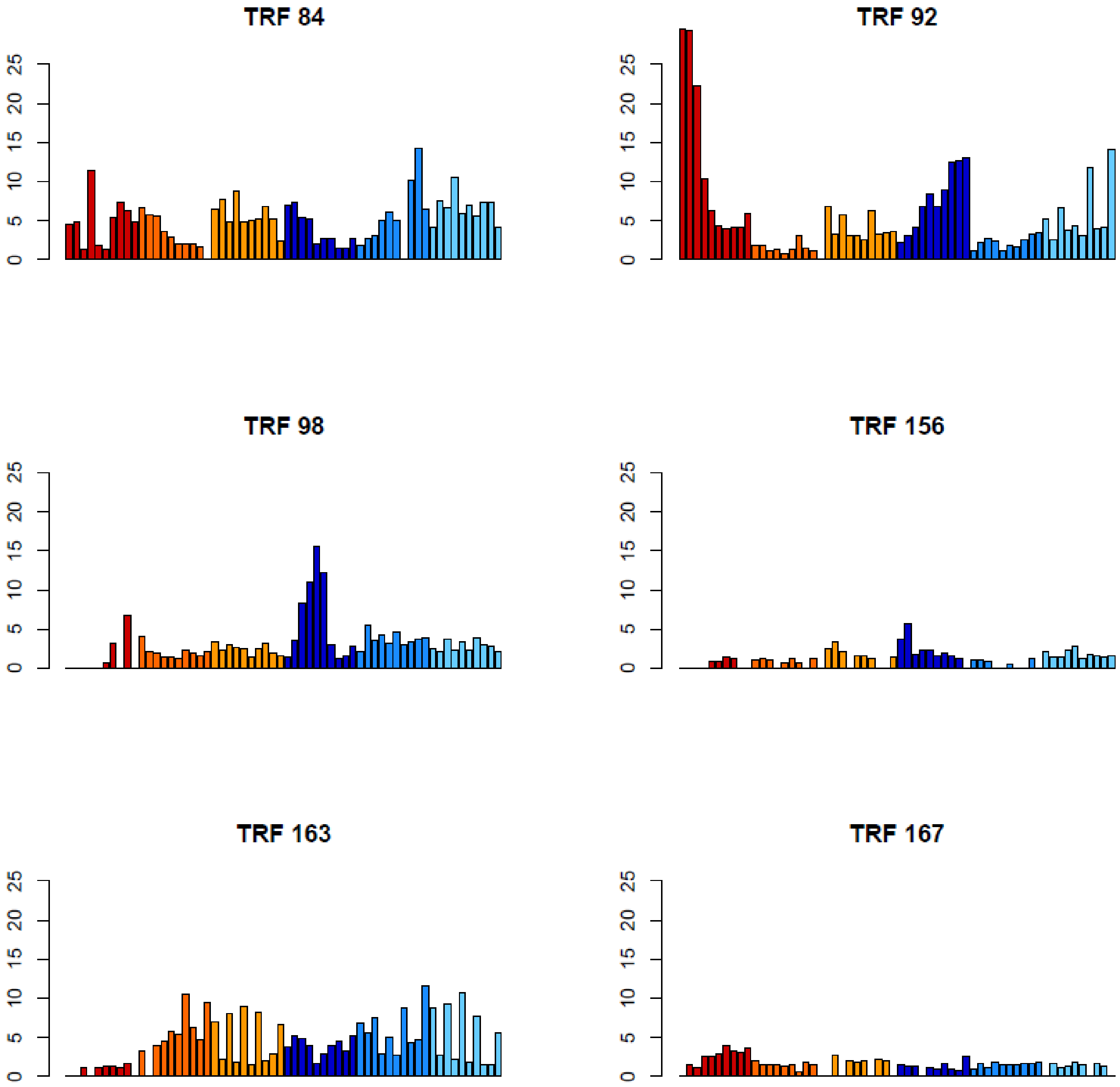

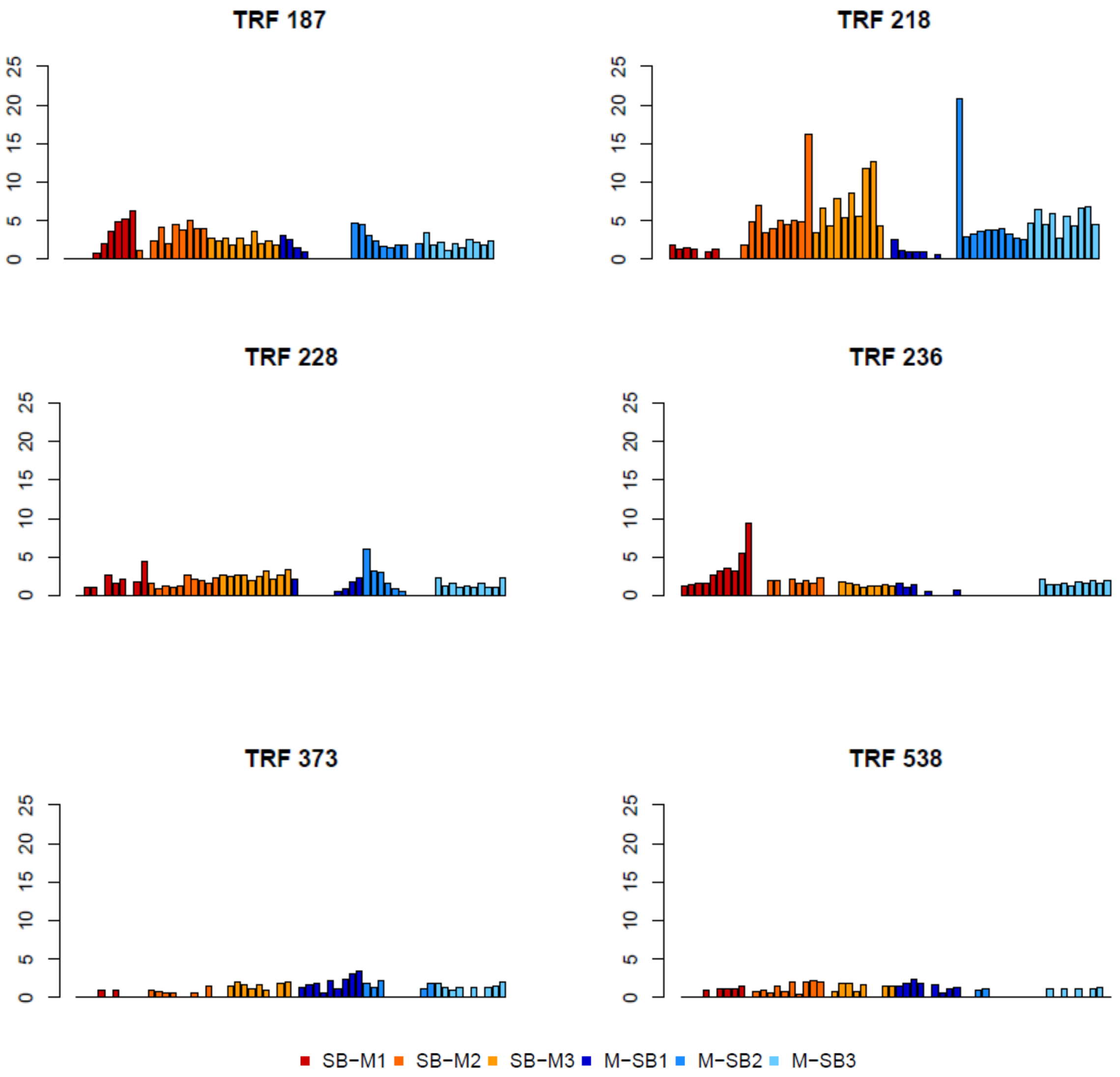

Figure S1. Relative abundance of the twelve terminal restriction fragments (TRFs) found in all six reactors at least at more than one time point.

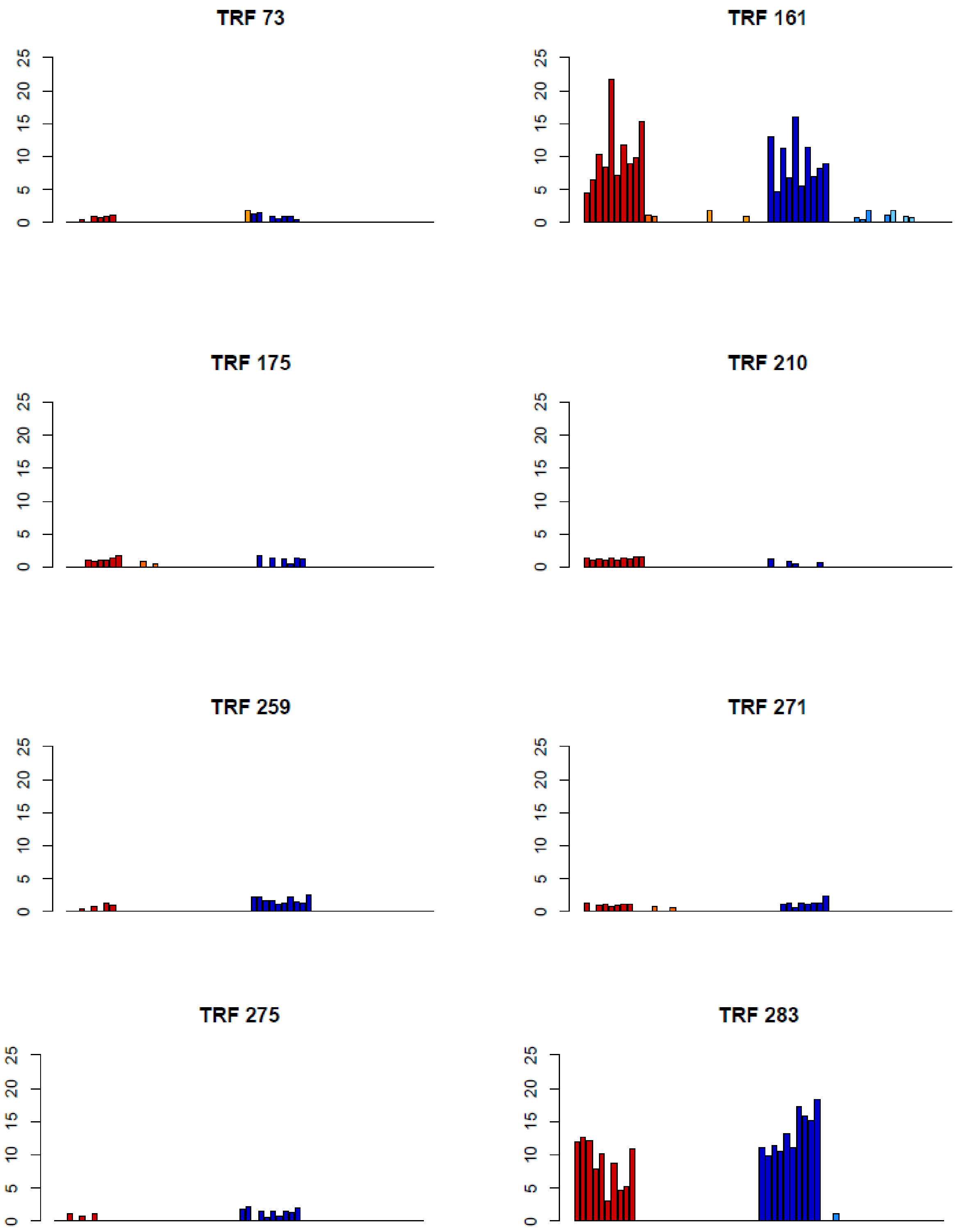

TRF 73
TRF 161
TRF 175
TRF 210
TRF 259
TRF 271
TRF 275
TRF 283
0 5 10 15 20 25

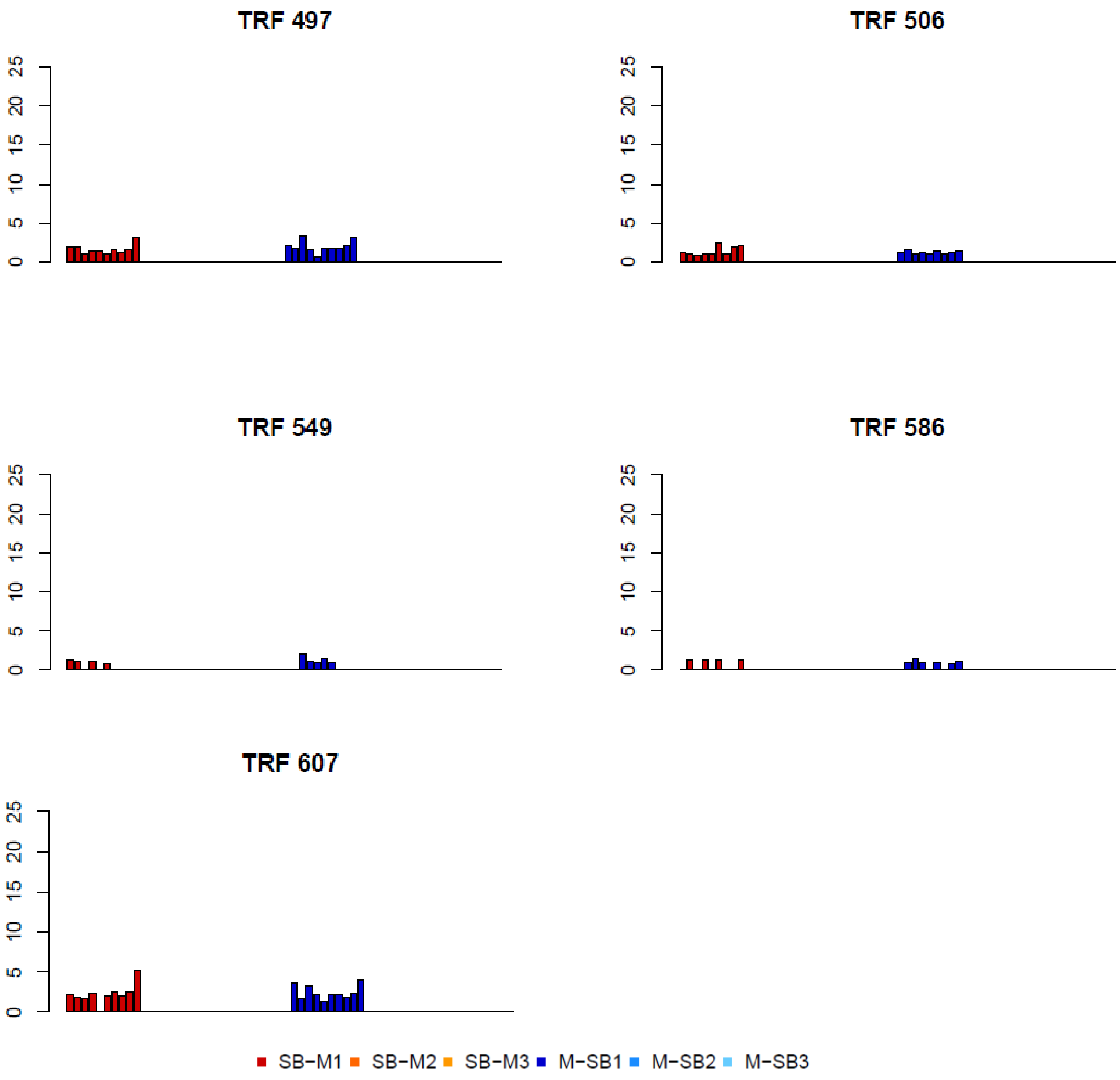

Figure S2. Relative abundance of the 13 terminal restriction fragments (TRFs) found mainly in the reactors with low TAN concentration.

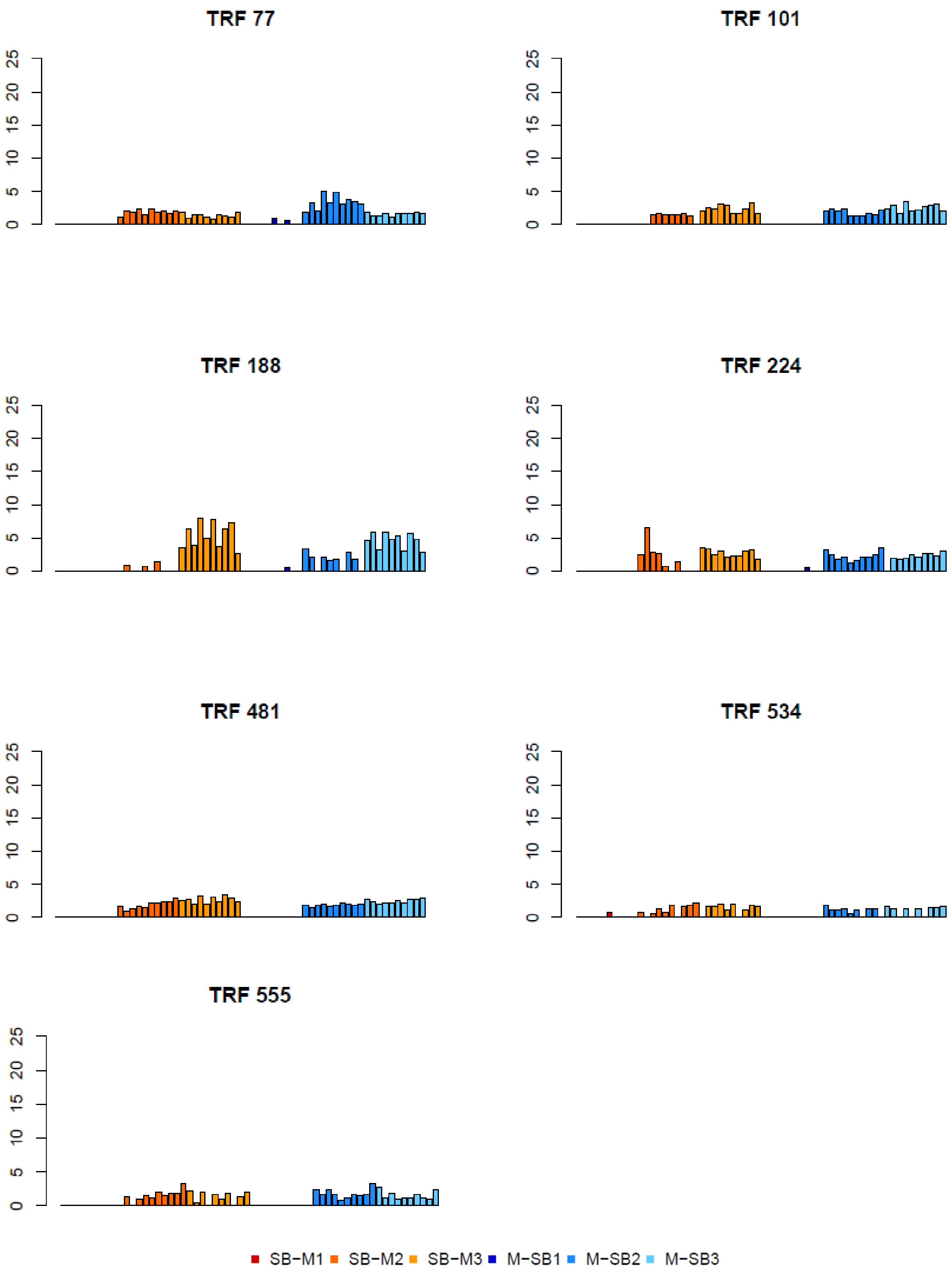

Figure S3. Relative abundance of the seven terminal restriction fragments (TRFs) found mainly in the reactors with high TAN concentration.

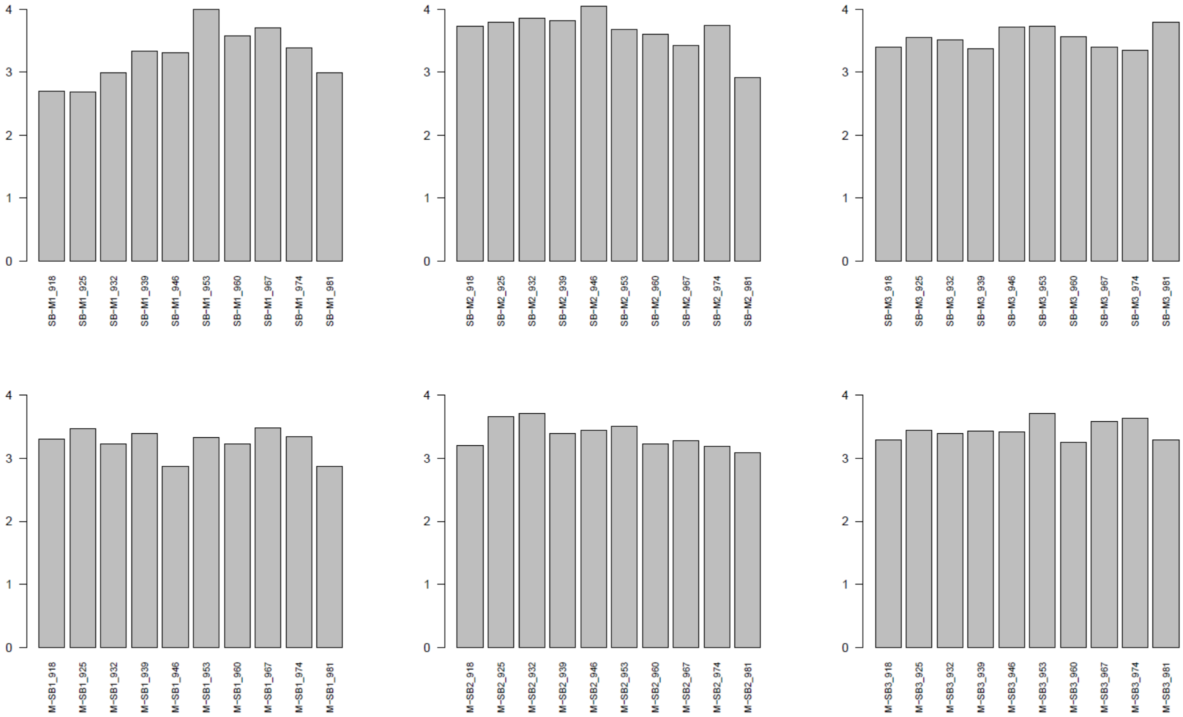

Figure S4. Shannon diversity indices for the recorded microbial communities within the six analysed reactors over the experimental phase.

Table S1. Indicator species analysis (ISA) in order to identify significant terminal restriction fragments (TRFs) for specific environmental conditions comparing the microbial community of all six reactors while grouping them into two groups: reactors with low and reactors with high total ammonium nitrogen (TAN) concentrations. The significance of each recorded TRF is provided by the calculated indicator value (IndVal) and a corresponding *p* value.

**ISA for Reactors with Low TAN**			**ISA for Reactors with High TAN**		
TRF	IndVal	*p* value	TRF	IndVal	*p* value
497	1.000	0.001	481	1.000	0.001
283	0.997	0.001	77	0.991	0.001
607	0.975	0.001	150	0.987	0.001
161	0.969	0.001	218	0.968	0.001
506	0.949	0.001	101	0.962	0.001
271	0.835	0.001	555	0.962	0.001
175	0.741	0.001	224	0.933	0.001
73	0.717	0.001	534	0.873	0.001
586	0.707	0.002			
230	0.688	0.002			
549	0.671	0.001			
96	0.500	0.042			

Table S2. Indicator species analysis (ISA) in order to identify significant terminal restriction fragments (TRFs) for specific environmental conditions comparing the microbial community of either the both reactors with low or the reactors with high total ammonium nitrogen (TAN) concentrations independently. The significance of each recorded TRF is provided by the calculated indicator value (IndVal) and a corresponding *p* value.

**ISA for SB-M1**			**ISA for M-SB1**			**ISA for Cluster I (SB-M2)**			**ISA for Cluster II (M-SB2)**			**ISA for Cluster III (SB-M3 & M-SB3)**		
TRF	IndVal	*p* value	TRF	IndVal	*p* value	TRF	IndVal	*p* value	TRF	IndVal	*p* value	TRF	IndVal	*p* value
296	0.949	0.001	518	0.986	0.001	285	0.863	0.001	554	0.798	0.001	135	0.837	0.001
236	0.928	0.001	384	0.949	0.003	532	0.722	0.001	104	0.793	0.001	108	0.824	0.001
210	0.888	0.001	98	0.923	0.003	165	0.707	0.002	79	0.775	0.002	334	0.766	0.003
146	0.853	0.005	163	0.915	0.001	216	0.684	0.007	579	0.600	0.009	377	0.671	0.008
88	0.837	0.003	259	0.913	0.001	75	0.684	0.004	143	0.553	0.019	63	0.631	0.015
128	0.837	0.002	373	0.900	0.003	146	0.632	0.016						
131	0.837	0.003	463	0.894	0.003	68	0.604	0.019						
375	0.837	0.002	156	0.867	0.003									
180	0.819	0.008	275	0.859	0.003									
370	0.803	0.015	546	0.853	0.003									
569	0.779	0.011	474	0.837	0.008									
394	0.775	0.015	502	0.776	0.014									
362	0.707	0.028	554	0.775	0.015									
521	0.707	0.030	527	0.722	0.019									
			294	0.721	0.020									
			445	0.707	0.034									
			550	0.662	0.047									
